# The influence of magnetic fields on the early ontogeny of the rainbow cichlid *Herotilapia multispinosa* (Günther, 1867)

**DOI:** 10.1186/s40851-026-00261-y

**Published:** 2026-02-03

**Authors:** Radosław Piesiewicz, Agata Korzelecka-Orkisz, Krzysztof Formicki

**Affiliations:** Department of Hydrobiology, Ichthyology and Biotechnology of Animal Reproduction, West Pomeranian University of Technology, Szczecin, Poland

**Keywords:** Magnetic field, Cichlids, Larval development, Embryogenesis, Larval growth, Survival rate, Deformities, Ornamental fish aquaculture

## Abstract

The aim of this study was to determine the influence of static magnetic fields with intensities of 1 mT, 3 mT, and 5 mT on the embryonic and postembryonic development of the rainbow cichlid (*Herotilapia multispinosa*). The experiments were conducted using eggs obtained from sexually mature, actively spawning pairs of this species. Analysis of the results showed that the applied magnetic fields significantly affected the rate of embryogenesis, which, depending on the intensity, could be either accelerated or slowed down. In particular, exposure to a 5 mT field accelerated development and produced larger hatchlings with smaller yolk sacs but lower subsequent growth, whereas exposure to a 3 mT field prolonged embryogenesis, resulting in smaller hatchlings with relatively larger yolk sacs and faster posthatching growth. The findings suggest that appropriately selected magnetic field parameters may positively influence the development of eggs and larvae of the rainbow cichlid, indicating the potential application of this method in ornamental fish aquaculture to reduce breeding losses.

## Introduction

The rainbow cichlid (*Herotilapia multispinosa*) is native to the freshwater habitats of Central America and is commonly found in Honduras, Nicaragua, and Costa Rica [1]. It prefers still waters and typically inhabits lakes and swamps with muddy substrates [2]. This species is among the smaller cichlids of Central America. Its body is oval or rounded, ending in a small terminal mouth equipped with characteristic tricuspid teeth. The dorsal fin bears small but sharp spines. The rainbow cichlid is distinguished by a yellow to honey-colored body and a distinct black stripe running from the head to the caudal fin. A circular spot is often visible in the middle of the body below the lateral line [1, 3].

Rainbow cichlids feed mainly on plant material, although studies suggest they may also display omnivorous feeding behavior. In addition to algae and plant fragments, numerous small fish scales have been found in their stomachs, and under laboratory conditions, they readily consumed *Artemia* and dipteran larvae [3]. These fish exhibit well-developed parental care: the female usually guards the eggs both day and night, while the male defends the nest [4].

Current evidence suggests that magnetic fields influence the early developmental stages of fish. Experimental studies on sea trout (*Salmo trutta*) and rainbow trout (*Oncorhynchus mykiss*) larvae have shown that magnetic fields affect body size in these species – exposure prolonged the incubation period, resulting in larvae that hatched longer and heavier [5]. Similarly, Krylov et al. [6] confirmed that magnetic fields affect both the size and weight of roach (*Rutilus rutilus*) larvae during incubation. Fey et al. [7] demonstrated that magnetic fields accelerate yolk sac resorption in northern pike (*Esox lucius*) larvae, with higher field densities corresponding to faster resorption rates.

Fey et al. [8] conducted experiments on rainbow trout (*O. mykiss*) larvae exposed to a magnetic field of 10 mT. Their results indicated no increase in body length or mass under these conditions; however, they confirmed an effect of the magnetic field on yolk sac resorption rate. Studies on green terror (*Andinoacara rivulatus*) larvae exposed to magnetic fields revealed reduced body size, larger yolk sacs, and smaller eyes compared to the control group. In contrast, exposure of Jaguar cichlid (*Parachromis managuensis*) larvae to magnetic fields affected both larval length and yolk sac dimensions, either increasing or decreasing them depending on the field density applied [9].

Scientific studies also suggest that magnetic fields can influence larval behavior. It has been shown that larvae of reef-dwelling fish use variations in magnetic field density during migration and for spatial orientation [10–13]. In haddock (*Melanogrammus aeglefinus*) and Atlantic cod (*Gadus morhua*) larvae, magnetic fields were found to negatively affect swimming speed and mean acceleration [14, 15]. Responses to magnetic fields have also been observed in Chinook salmon (*Oncorhynchus tshawytscha*), whose larvae, upon leaving the hatching site, moved in the direction of the generated magnetic field [16]. Myklatun et al. [17] demonstrated the effect of magnetic fields on five-day-old Medaka (*Oryzias latipes*) larvae, whose response was characterized by increased swimming activity during exposure to the magnetic fields.

Studies on rainbow trout (*Oncorhynchus mykiss*) have demonstrated a significant effect of magnetic fields on egg fertilization efficiency [18]. Sea trout (*Salmo trutta*) eggs exposed to magnetic fields of varying density (1–5 mT) showed an increase in volume compared with the control group [19]. Similar, though less pronounced, effects were observed in vendace (*Coregonus albula*) eggs [19]. Magnetic fields may also influence the early stages of ontogeny – from ectoplasmic movement within the egg cell and rotation of the germinal disc, through orientation responses (embryos aligning with spatial stimuli), to cardiac contractions and somatic movements [20]. They can also affect cardiac activity by increasing heart rate with rising field density [21].

Research on Jaguar cichlid (*Parachromis managuensis*) embryos has shown that magnetic fields of 1 mT and 3 mT slightly shorten the course of embryogenesis [22]. Comparable results were obtained for pike (*Esox lucius*) eggs exposed to magnetic fields – their incubation period was shorter, and development occurred faster than in the control group [7]. Magnetic fields have also been shown to affect the spatial orientation of embryos in several fish species, causing them to align parallel to either natural or artificially generated magnetic field lines [5, 23–28]. In addition, magnetic fields may increase the number of melanophores on the surface of developing embryos [29].

Magnetic fields can also influence egg survival. In Jaguar cichlids, higher egg survival rates were recorded compared to the control group [22]. However, studies on pike (*E. lucius*) and rainbow trout (*O. mykiss*) eggs found no significant effect of magnetic fields on egg survival [7, 8].

Despite the growing body of research on the effects of magnetic fields on fish, information on their influence on egg and larval behavior remains limited and fragmented. To date, most studies have focused on species used in large-scale aquaculture [30], while data on ornamental fish are still lacking – particularly for the widely popular cichlid family [31]. No studies have yet examined the effects of magnetic fields on the egg and larval development of the rainbow cichlid (*Herotilapia multispinosa*).

The aim of this study was to describe in detail the ontogeny of the rainbow cichlid (*H. multispinosa*) and to investigate the effects of magnetic fields on its eggs and larvae. Specifically, the research focused on evaluating the impact of magnetic fields with intensities of 1 mT, 3 mT, and 5 mT on embryonic development time, egg size, heart activity (heart rate per minute), and egg survival. During the larval stage, attention was given to the effects of magnetic fields on body and eye size, yolk sac resorption rate, survival rate, and the occurrence of deformities. The study also aimed to determine whether magnetic fields exert beneficial or adverse effects on larvae of this species, which could, in the future, support the use of magnetic fields in ornamental fish aquaculture to reduce losses during both the larval and early juvenile stages.

## Materials and methods

The experiments were conducted under controlled laboratory conditions in an isothermal facility, where temperature was carefully maintained to ensure optimal conditions for egg development and hatching. The study was performed on the eggs and larvae of the rainbow cichlid (*H. multispinosa*).

### Egg incubation stage

From five spawning pairs of rainbow cichlid, 1200 fertilized eggs were collected from each pair in a single sampling. The eggs were divided into four equal portions of 300 each and placed in separate 500 ml glass containers. All containers were filled with water taken directly from the spawning aquarium to maintain stable and as natural physicochemical conditions as possible.

**Fig. 1:**
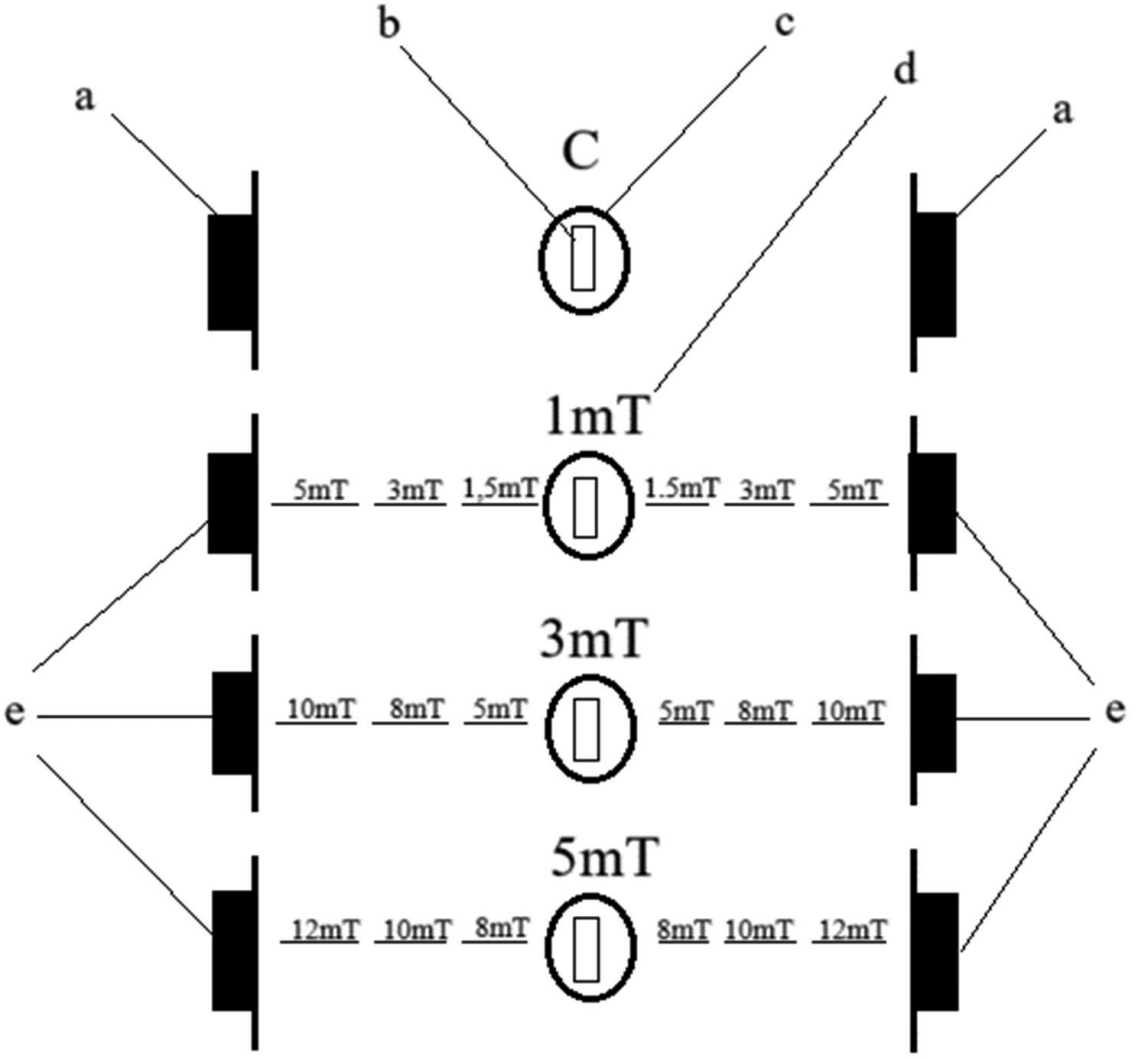
Diagram of the experiment showing the arrangement of the magnetic field values during this study. The magnetic fields between the individual trials did not interfere with each other. The letters represent the following: a – magnet dummy, b – location of the eggs/larvae exposed to the magnetic field, c – crystallizer, d – tested magnetic field value affecting the eggs/larvae, and e – magnets generating the magnetic field

Each container was adequately aerated, and a 25 W heater maintained a constant water temperature of 26 ± 0.5 °C. Before incubation began, 300 eggs were transferred into each container using a 2 ml pipette. The containers were arranged so that the magnetic field generated by magnets placed on their sides acted on the eggs with a specific density. The control group (C) was not exposed to the magnetic field – only dummy magnets were used. In the experimental variants, magnetic fields of 1 mT (Trial 1), 3 mT (Trial 2), and 5 mT (Trial 3) were applied, corresponding to densities approximately 20, 60, and 100 times stronger than the Earth’s magnetic field. It is worth noting that the static magnetic fields examined in this study are several times stronger than those affecting fish in their natural environment due to submarine power cables [32] or high-voltage transmission lines [27]. The magnetic field induction for each configuration was measured using a magnetic field meter (Fig. 1).

#### Observation of embryonic development

Embryonic development was monitored continuously from fertilization to the hatching stage. At regular intervals, five eggs were randomly selected from each group for microscopic observation. Observations were conducted using a light microscope (Nikon 2000SE, Tokyo, Japan) and a stereomicroscope (Carl Zeiss Stereo Discovery V12, Jena, Germany), both equipped with software for digital image recording.

During the early phases of embryogenesis – from fertilization to the onset of gastrulation, which occurred approximately 8–9 hours post-fertilization depending on the experimental batch – photographic documentation was performed every 15 minutes. In later stages, due to slower developmental progression, observations were made every two hours until hatching. All observations were conducted on Petri dishes using randomly selected samples.

During organogenesis, heart activity of developing embryos was also measured. The heart rate (beats per minute) was recorded three times: immediately after the onset of cardiac activity, midway through organogenesis, and at its completion. For each measurement, 15 randomly selected eggs from each group were used (a total of *n* = 375 measurements from 25 spawnings). The obtained data were averaged, and statistical differences between groups were analyzed using Scheffé’s tests.

The duration of individual stages of embryonic development was expressed in hours post-fertilization (hpf) and converted into degree-hours (°H), defined as the product of incubation time (in hours) and the mean water temperature.

Dead eggs were removed daily from the glass containers to prevent interference with the experiment. The number of removed eggs was recorded throughout the study, and egg survival (Pj) was calculated using the following formula:

$${\bf{Pj}}\left[ \% \right] = \left({{\bf{a}} \times 100} \right)/{\bf{b}}$$

where *a* – number of dead eggs, *b* – total number of eggs in the tested group.

### Larval stage analysis

After hatching, larval development was observed twice daily for three consecutive days, until the yolk sac had resorbed to approximately 30% of its original volume. Each day, at the same time, 11 individuals were randomly selected from each container. The larvae were subjected to detailed morphometric analysis and then removed to avoid repeated handling and minimize stress effects.

Survival rate (Ls) was calculated using the following formula:

$${\bf{Ls}}\left[ \% \right] = \left({{\bf{a}} \times 100} \right)/{\bf{b}}$$

where *a* – number of dead larvae, *b* – total number of larvae in the examined group.

The number of dead and deformed larvae was recorded daily. The percentage of deformities (Ld) was determined according to the formula:

$${\bf{Ld}} \left[ \% \right] = \left({{\bf{a}} \times 100} \right)/{\bf{b}}$$

where *a* – number of larvae exhibiting visible deformities, *b* – total number of individuals in the group. Morphological criteria used to assess deformities included body malformations, head deformities, and yolk sac abnormalities.

**Fig. 2:**
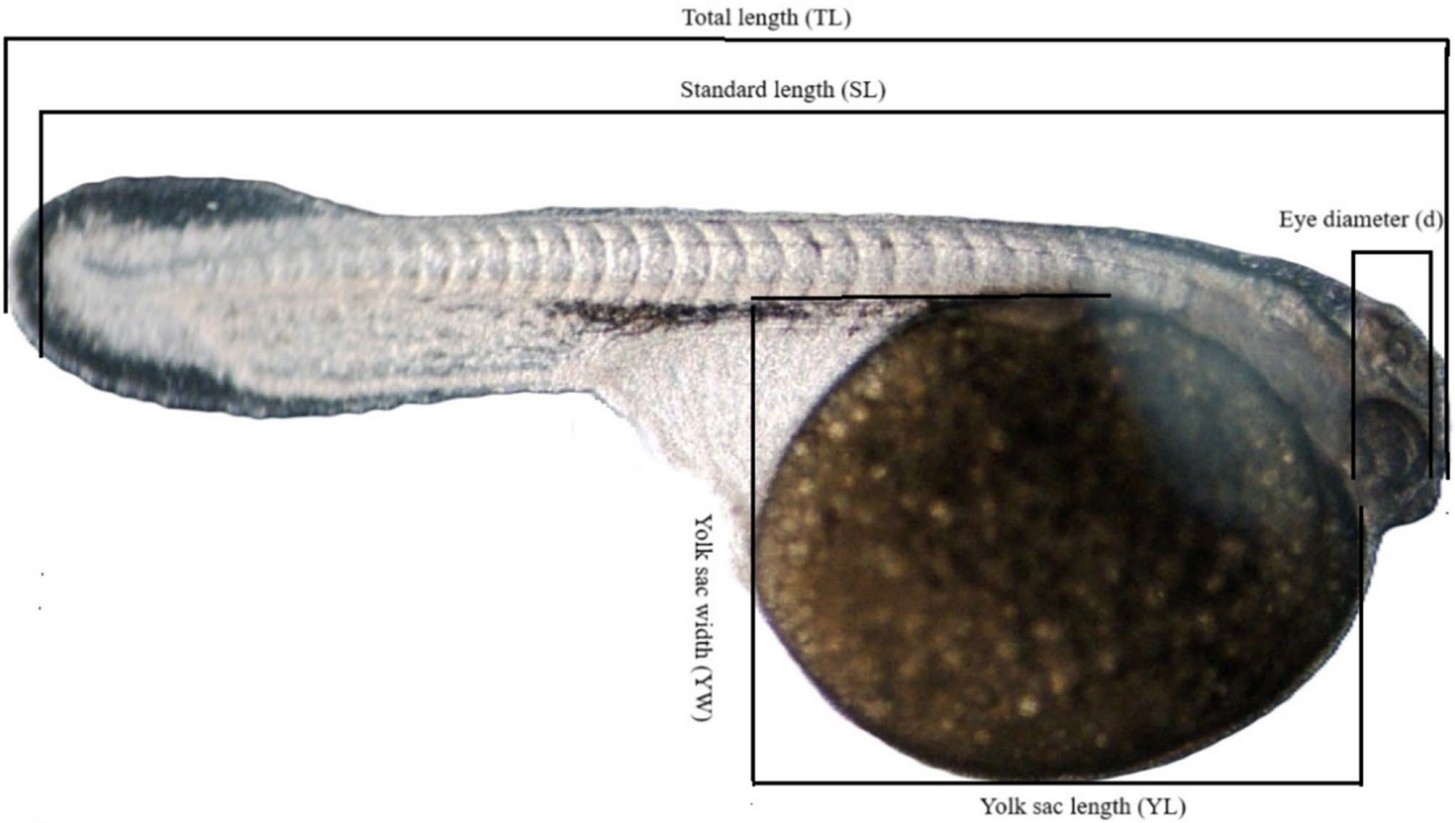
Jaguar cichlid larva with labels: total length (TL), standard length (SL), yolk sac length (YL), yolk sac width (YW) and eye diameter (d)

At the end of the observation period, detailed biometric measurements were taken using Multiscan software. The following parameters were recorded: total length (TL), standard length (SL), yolk sac length (YL) and width (YW), and eye diameter (d). Yolk sac volume was calculated using the formula for an elongated ellipsoid (Fig. 2):

$${\bf{V}} = \left({{\bf{\pi }}/6} \right) \times {\bf{YL}} \times {\bf{YW}}2$$

The collected data were subjected to statistical analysis using Statistica software (version 13.3). Data normality was assessed using the Shapiro–Wilk test, while homogeneity of variances was evaluated with Levene’s test. After confirming that the assumptions for one-way analysis of variance (ANOVA) were met, mean values were compared using Scheffé’s post hoc test. Differences were considered statistically significant at *p* < 0.05. The results are presented in the text as mean ± standard deviation (SD), while in the graphs, the points represent the mean and the “whiskers” indicate the standard deviation.

## Results

### Embryonic development

The course of embryogenesis and the timing of its key stages are presented in Table 1.

**Table 1:** Duration of embryogenesis in the rainbow cichlid *H. multispinosa*. The symbol “C” denotes the control group, while 1 mT, 3 mT, and 5 mT represent the tested magnetic field densities

Rainbow cichlid		C		1mT		3mT		5mT	
		Degree hour (°H)	Hour (Hpf)	Degree hour (°H)	Hour (Hpf)	Degree hour (°H)	Hour (Hpf)	Degree hour (°H)	Hour (Hpf)
Fertilization		0	0	0	0	0	0	0	0
Cleavage	2 blastomeres	26	1	26	1	26	1	26	1
	4 blastomeres	34.5	1.33	34.5	1.33	34.5	1.33	34.5	1.33
	8 blastomeres	45,5	1.75	45,5	1.75	45,5	1.75	45,5	1.75
	16 blastomeres	54	2.08	54	2.08	54	2.08	54	2.08
Gastrulation	beginning	208	8	208	8	234	9	208	8
	epiboly 1/3	260	10	260	10	351	13,5	260	10
	epiboly ½	351	13.5	351	13.5	429	16.5	351	13.5
	3/4 epiboly	429	16.5	429	16.5	494	19	429	16.5
	Closure of the blastopore	468	18	468	18	533	20.5	468	18
Organogenesis	Making the head part visible	494	19	494	19	546	21	481	18.5
	Eye primordia	507	19.5	507	19.5	559	21.5	494	19
	First heart contractions	728	30	780	28	806	31	624	24
	First movements of the embryo	949	36.5	975	37.5	1027	39.5	780	30
	Making the brain visible	975	37.5	1001	38.5	1040	40	806	31
	Pigment cells become visible in the lens	1001	38.5	1027	39.5	1079	41.5	858	33
	The appearance of pigment cells on the body	1027	39.5	1053	40.5	1105	42.5	884	34
Hatching	First hatching	1092	43	1118	42	1196	46	936	36

### Cleavage

The initial analysis of eggs collected from spawning pairs of rainbow cichlids showed no differences in the quality of the material. Observations of the first phase of embryonic development – cleavage – revealed no visible differences between the experimental and control groups. In all groups, 2 blastomeres appeared at 26 °H, 4 blastomeres at 34.5 °H, 8 blastomeres at 45.5 °H, and 16 blastomeres at 54 °H. Cleavage ended when the embryos reached the blastula stage, characterized by the presence of an epiblast and a hypoblast layer covering the yolk sphere, with a cavity – the blastocoel – between them.

### Gastrulation

Differences in the rate of developmental progression were first observed during the gastrulation stage. The onset of gastrulation occurred at 208 °H in the control group and in trials 1 (1mT) and 3mT), while in trial 2 (3mT) it was recorded latest, at 234 °H (Table 1). The stage of one-third yolk sphere coverage (epiboly ⅓) was observed at 260 °H in the control, 1 mT, and 5 mT groups, but 91 °H later in the 3 mT group. Half coverage (epiboly ½) was reached at 351 °H in the control, 1 mT, and 5 mT groups, and 78 °H later in the 3 mT group. Three-quarter coverage (epiboly ¾) was also recorded latest in trial 2 (3mT). The end of gastrulation, corresponding to complete yolk coverage (blastopore closure), was likewise observed latest in the 3mT group, at 533 °H. In all other experimental and control groups, this stage was completed approximately 65 °H earlier (Table 1).

### Organogenesis

In the rainbow cichlid, the first visible outline of the embryo – particularly the developing head region – appeared earliest in trial 3 (5mT), followed by the control and trial 1 (1mT), and lastly in trial 2 (3mT). These differences in the rate of organ formation persisted until hatching. Eye primordia were first observed in trial 3 (5mT), then in the control and 1 mT groups, and finally in the 3 mT group. The first embryonic movements were noted earliest in trial 3 (5mT), followed by the control group, trial 1 (1mT) at 975 °H, and lastly trial 2 (3mT). Developing brain vesicles appeared first in trial 3 (5mT), followed by the control, trial 1 (1mT), and trial 2 (3mT). Pigmentation in the lens appeared earliest in trial 3 (5mT) at 858 °H, then in the control group at 1001 °H, in trial 1 (1mT) at 1027 °H, and in trial 2 (3mT) at 1079 °H. Body pigmentation appeared first in trial 3 (5mT), followed by the control group, trial 1 (1mT) at 1053 °H, and lastly trial 2 (3mT) (Table 1).

### Heart

Heart contractions in rainbow cichlid embryos were first observed in trial 3 (5mT) at 624 °H, followed by the control group at 728 °H, trial 1 (1mT) at 780 °H, and finally trial 2 (3mT) at 806 °H. Embryos from different experimental groups exhibited varying heart rates. The highest rate was recorded in embryos from trial 2 (3mT) − 37 beats per minute, followed by the control group − 36 beats per minute, and trial 3 (5mT) − 35 beats per minute. The lowest rate was observed in embryos from trial 1 (1mT) − 33 beats per minute. However, statistical analysis showed that these differences were not statistically significant among the groups (Scheffé post hoc test, *p* > 0,11).

**Fig. 3:**
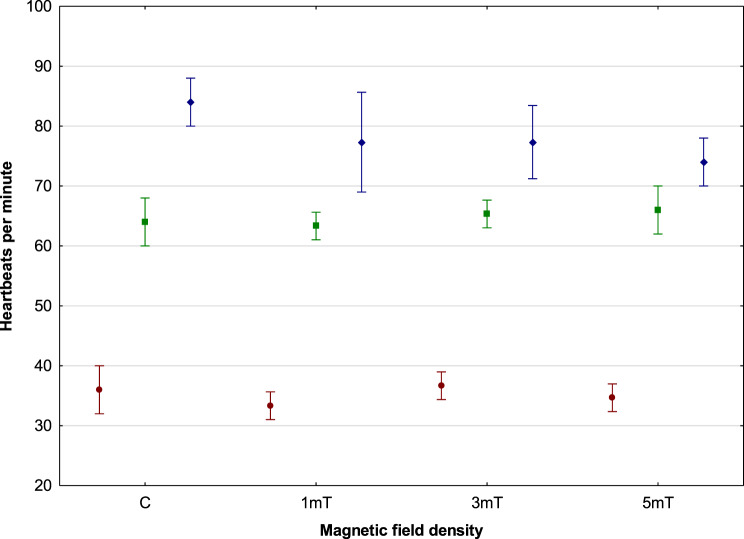
Heart rate (beats per minute) in the rainbow cichlid (*H. multispinosa*). Measurement 1 (after the onset of cardiac activity) is shown in red, measurement 2 (mid-organogenesis) in green, and measurement 3 (end of organogenesis) in blue (*n* = 375)

In all experimental variants, heart development followed a similar pattern: initially, slow and irregular contractions were observed, which became progressively more regular as the embryos developed and the heart rate increased. By the end of embryogenesis, the heartbeat was steady and rhythmic. In the control group, the heart rate reached 84 beats per minute. Slightly slower heart activity was recorded in trials 1 (1mT) and 2 (3mT) − 77 beats per minute – while the slowest rate was observed in trial 3 (5mT) − 74 beats per minute. No statistically significant differences were found between the control group and trials 1 (1mT) or 2 (3mT). However, the difference in heart rate between the control group and trial 3 (5mT) was statistically significant (*p* = 0.021). No statistically significant differences were found among trials 1 (1mT), 2 (3mT), and 3 (5mT), (Scheffé post hoc test, *p* > 0,12) (Fig. 3).

## Embryo survival and hatching

**Fig. 4:**
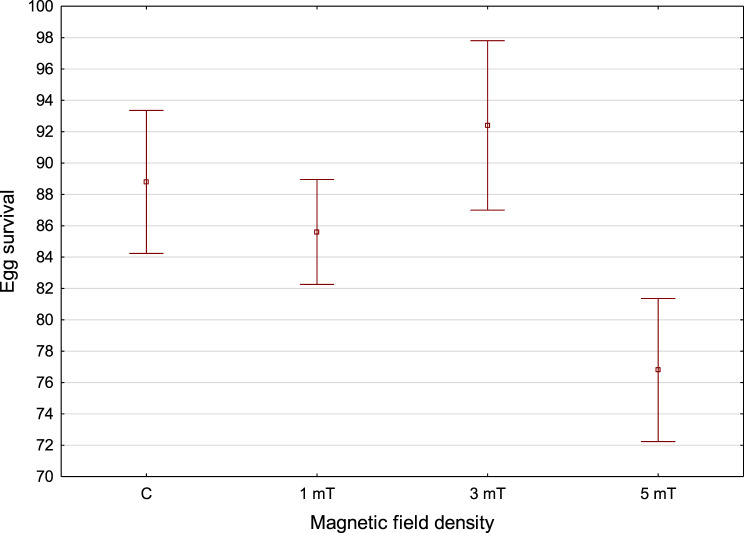
Egg survival of the rainbow cichlid (*H. multispinosa*) in the following groups: C – control, trial 1–1 mT, trial 2–3 mT, trial 3–5 mT

The highest egg survival was observed in group 2 (3mT) − 92%. In the control group, egg survival reached 89%. Slightly lower egg survival was recorded in group 1 (1 mT) at 86%. The lowest egg survival was observed in group 3 (5 mT), amounting to 77% (Fig. 4).

Hatching occurred earliest in trial 3 (5mT), followed by the control group at, and 26 °H later in trial 1 (1mT). The latest hatching was observed in trial 2 (3mT. Hatching in all groups occurred over an extended period. In trial 3 (5mT), 50% of larvae hatched at 1066 °H, and complete hatching was recorded at 1196 °H. In the control, 1mT, and 3mT groups, 50% hatching occurred 156 °H later, with full hatching achieved after an additional 130 °H.

### Larval size

**Fig. 5:**
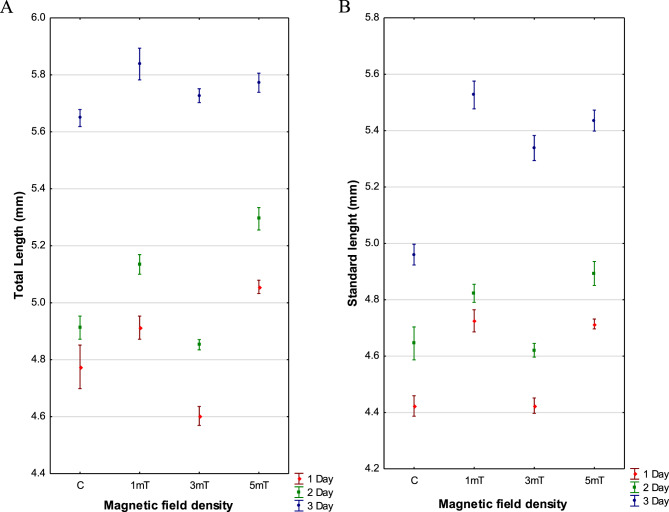
Total length (**A**) and standard length (**B**) of rainbow cichlid (*H. multispinosa*) larvae during the first three days post-hatching. Day 1 – *n* = 2375, day 2 – *n* = 2100, day 3 – *n* = 1825

The largest newly hatched larvae were observed in trial 3 (5mT), with an average total length of 5.056 ± 0.012 mm and an average standard length of 4.714 ± 0.009 mm. Slightly smaller larvae were recorded in trial 1 (1mT), with an average total length of 4.913 ± 0.020 mm and a standard length of 4.725 ± 0.020 mm. In the control group (not exposed to a magnetic field), the mean total length was 4.775 ± 0.038 mm, and the mean standard length was 4.423 ± 0.018 mm. The smallest larvae were found in trial 2 (3mT), with an average total length of 4.603 ± 0.017 mm and a standard length of 4.424 ± 0.014 mm. The Scheffé post hoc test revealed significant differences among all examined experimental variants (*p* < 0.001) (Fig. 5 A, B).

Measurements taken on the third (final) day of observation showed variation in larval growth among the groups. The largest larvae were recorded in trial 1 (1mT), with a total length of 5.838 ± 0.028 mm and a standard length of 5.526 ± 0.025 mm. Slightly smaller larvae were observed in trial 3 (5mT), with a total length of 5.772 ± 0.017 mm and a standard length of 5.436 ± 0.019 mm. Larvae from trial 2 (3mT) were somewhat smaller, with a total length of 5.727 ± 0.012 mm and a standard length of 5.338 ± 0.022 mm. The smallest larvae were found in the control group, with a total length of 5.648 ± 0.015 mm and a standard length of 4.960 ± 0.018 mm. The Scheffé post hoc test revealed significant differences among all examined experimental variants (*p* < 0.001).

Changes in larval body growth were also observed during the first three days post-hatching. The highest growth increment was recorded in trial 2 (3mT) − 1.124 mm. In trial 1 (1mT), larval growth reached 0.925 mm, while slightly lower growth was noted in the control group (0.873 mm). The smallest growth increment was observed in trial 3 (5mT) − 0.712 mm.

### Yolk sac

**Fig. 6:**
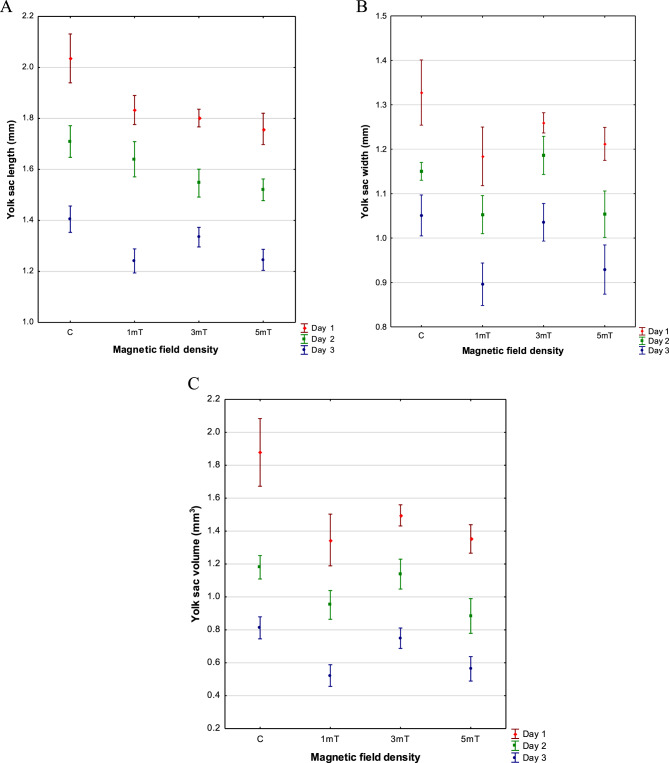
Length (**A**), width (**B**), and volume (**C**) of the yolk sac in rainbow cichlid (*H. multispinosa*) larvae. Day 1 – *n* = 2375, day 2 – *n* = 2100, day 3 – *n* = 1825

The largest yolk sac in rainbow cichlid larvae was observed in the control group, with mean dimensions of YL = 2.035 ± 0.048 mm, YW = 1.328 ± 0.037 mm, and V = 1.878 ± 0.103 mm^3^. Smaller yolk sacs were found in larvae from trial 2 (3mT), measuring on average YL = 1.802 ± 0.017 mm, YW = 1.259 ± 0.011 mm, and V = 1.495 ± 0.032 mm^3^. In larvae exposed to a magnetic field of 1mT (trial 1), the yolk sac measured YL = 1.834 ± 0.027 mm, YW = 1.184 ± 0.033 mm, and V = 1.346 ± 0.079 mm^3^. The smallest yolk sacs were recorded in larvae from trial 3 (5mT), with mean dimensions of YL = 1.759 ± 0.031 mm, YW = 1.212 ± 0.019 mm, and V = 1.352 ± 0.043 mm^3^ (Fig. 6 A,B,C).

This pattern of differences in yolk sac size, reflecting the rate of yolk resorption among larvae from the various experimental groups, was maintained throughout the first three days of larval development. On the third day, the largest yolk sacs were again observed in control larvae, with mean dimensions of YL = 1.405 ± 0.026 mm, YW = 1.051 ± 0.023 mm, and V = 0.812 ± 0.034 mm^3^. Compared with the yolk sac volume at hatching, this represented 57% resorption. In trial 2 (3mT), the yolk sac measured YL = 1.335 ± 0.019 mm, YW = 1.035 ± 0.021 mm, and V = 0.749 ± 0.031 mm^3^, corresponding to 50% resorption. Larvae from trial 3 (5mT) had yolk sacs with mean dimensions of YL = 1.245 ± 0.021 mm, YW = 0.925 ± 0.028 mm, and V = 0.563 ± 0.037 mm^3^, representing 58% of the original yolk sac volume. The smallest yolk sacs were recorded in larvae exposed to a magnetic field of 1mT (trial 1), with YL = 1.241 ± 0.024 mm, YW = 0.896 ± 0.024 mm, and V = 0.522 ± 0.033 mm^3^, corresponding to 61% resorption.

No correlation was observed between larval growth rate and yolk sac resorption rate in rainbow cichlid larvae.

### Eyes

**Fig. 7:**
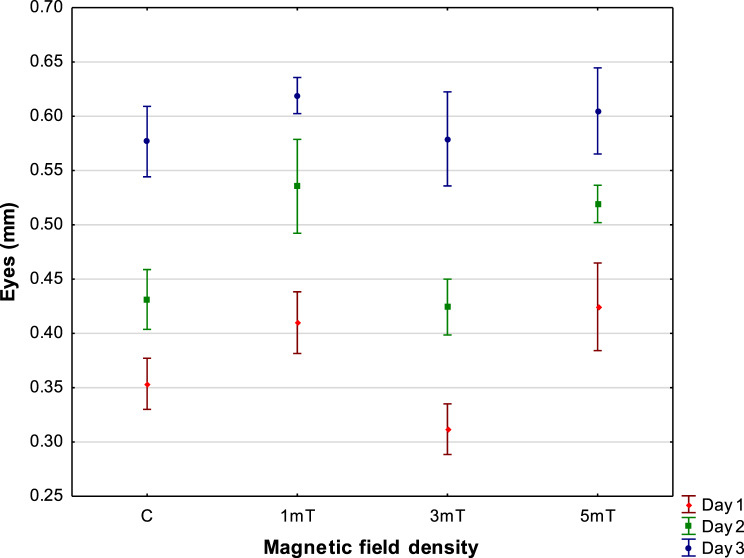
Changes in eye diameter of rainbow cichlid (*H. multispinosa*) larvae. Day 1 – *n* = 2375, day 2 – *n* = 2100, day 3 – *n* = 1825

Measurements of eye diameter in rainbow cichlid larvae revealed considerable variation among experimental groups exposed to different magnetic field densities. The largest eyes were recorded in newly hatched larvae from trial 3 (5mT), with an average diameter of 0.425 ± 0.020 mm. Slightly smaller eyes were observed in larvae from trial 1 (1mT), with an average diameter of 0.410 ± 0.014 mm. In the control group, mean eye diameter measured 0.354 ± 0.012 mm, while the smallest eyes were found in larvae from trial 2 (3mT), with an average diameter of 0.312 ± 0.012 mm (Fig. 7).

Measurements taken after three days of larval development showed that eye growth was correlated with overall body growth. The largest eyes were recorded in larvae from trial 1 (1mT), with a mean diameter of 0.619 ± 0.008 mm. Slightly smaller eyes were observed in larvae from trial 3 (5mT) − 0.601 ± 0.008 mm. Larvae from the control and 3mT groups had nearly identical eye sizes, with average diameters of 0.557 ± 0.010 mm and 0.579 ± 0.022 mm, respectively.

### Larval survival and deformities

**Fig. 8:**
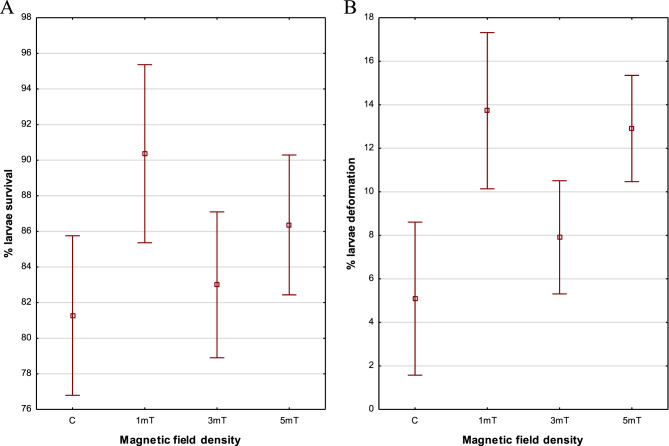
Survival rate (**A**) and percentage of deformities (**B**) in rainbow cichlid (*H. multispinosa*) larvae

The results of the experiment indicate that magnetic fields affect the survival of rainbow cichlid larvae. After three days, the highest larval survival rate was recorded in trial 1 (1mT) − 90%. Slightly lower survival was observed in trial 3 (5mT) − 86%, followed by trial 2 (3mT) − 83%. The lowest survival rate was found in the control group (81%), which was not exposed to a magnetic field. No statistically significant difference was found between the control group and group 2 (3mT) using the Scheffé post hoc test (*p* = 0.348). The Scheffé post hoc test revealed significant differences among the remaining experimental variants (*p* < 0.001) (Fig. 8 A).

Analysis of larval deformities showed that the percentage of deformed larvae depended on the density of the magnetic field applied throughout embryogenesis. Body deformities included scoliosis of varying severity and yolk sac malformations. The highest proportion of deformities (14% of all individuals) occurred in larvae from trial 1 (1mT), followed by trial 3 (5mT) with 13% deformed individuals. In trial 2 (3mT), 8% of larvae exhibited body deformities, while the control group showed the lowest incidence, with only 5% of individuals affected. The Scheffé post hoc test revealed no significant difference between groups 1 and 3 (*p* = 0.672), whereas comparisons of the other experimental variants using the Scheffé post hoc test showed statistically significant differences (*p* < 0.001) (Fig. 8 B).

## Discussion

The conducted experiment expands current knowledge on the effects of magnetic fields of selected densities (1mT, 3mT, and 5mT) on the eggs and larvae of the rainbow cichlid. The results indicate that magnetic fields can exert both positive and negative effects on eggs during embryogenesis and on larvae during the early stages of development. This study focused on assessing the influence of magnetic fields on egg size, the rate of embryonic development, heart rate dynamics, and embryo survival. Subsequent observations also examined the effects of magnetic fields on larval body size, eye diameter, yolk sac volume, as well as survival rate and the occurrence of body deformities.

The number of studies investigating the effect of magnetic fields on the duration of embryogenesis remains limited; however, existing data suggest that both magnetic and electromagnetic fields significantly affect fish embryonic development. For certain species, such as rainbow trout (*O. mykiss*), brown trout (*S. trutta*), and northern pike (*E. lucius*), the influence of magnetic fields on egg incubation has been documented [23, 24, 27, 28, 30, 33]. In studies on pike (*E. lucius*), magnetic field exposure was found to accelerate embryogenesis, with eggs developing faster than those in the control group [7].

Analysis of the results obtained in this experiment showed that the effects of the magnetic field were diverse and depended both on the developmental stage of the rainbow cichlid and on the field density. No significant effect of the magnetic field was observed during the cleavage stage. The likely reason is the short duration of this phase in the studied species, which resulted in too brief an exposure time for the eggs to the magnetic field. Pronounced differences in developmental dynamics under magnetic field exposure were observed only in the subsequent stages of embryogenesis, with the effects depending on field strength. During gastrulation, a slowdown in egg development was noted in the group exposed to a magnetic field of 3mT, and this effect persisted throughout later developmental stages. In contrast, eggs exposed to a 5mT magnetic field developed the fastest. In the group exposed to the weakest field density (1mT), development was slower than in the control group. Very similar effects were reported in studies on the orange-fin cichlid (*A. rivulatus*) [9], where exposure to a 5mT magnetic field accelerated egg development, while lower field densities (1mT and 3mT) slowed it down. Conversely, in experiments on the Jaguar cichlid (*P. managuensis*), egg development was faster under exposure to magnetic fields of 1mT and 3mT [9].

Differences in the developmental rate of the rainbow cichlid were correlated with embryo survival. Faster development in trial 3 (5mT) resulted in considerably lower egg survival, whereas slower development in trial 2 (3mT) led to a 15% higher survival rate. In contrast to the present findings, studies on rainbow trout (*O. mykiss*) eggs exposed to a 10mT magnetic field showed no statistically significant effect of the field on egg survival [7, 8].

Changes in magnetic field density significantly affect cardiac activity dynamics. In experiments conducted by Winnicki et al. [34] and Korzelecka-Orkisz et al. [21], exposure to a magnetic field caused an increase in heart rate during the first few minutes (5–10 min) of exposure, after which the frequency of heart contractions generally returned to pre-stimulus levels. The results of the present study showed that embryonic hearts also responded to magnetic field exposure, although in a different way – heart rate decreased. This effect may be attributed to the design of the experiment, as the embryos were continuously exposed to the magnetic field and then removed from its influence.

Experiments conducted on the rainbow cichlid indicate that the magnetic field affected not only embryos during embryogenesis but also individuals in the subsequent developmental stage (larvae), resulting in differences in growth rate. The magnetic field influenced the rate of development and consequently larval size. Larvae exposed to a 5mT magnetic field – the variant in which embryogenesis progressed the fastest – hatched as the largest individuals and had the smallest yolk sacs. However, exposure to this field density resulted in the lowest body growth during the following days of development. In contrast, larvae from trial 2 (3mT), which had the longest embryogenesis, hatched as the smallest and possessed the second-largest yolk sacs. Exposure to this field density promoted the fastest body growth during the first days of larval life.

Research on other fish species shows that the effects of magnetic fields vary depending on the species. Piesiewicz et al. [9] demonstrated that magnetic fields affect larval size in the orange-fin cichlid (*A. rivulatus*). Larvae of this species exposed to magnetic fields of 1mT, 3mT, and 5mT were smaller than those not subjected to magnetic influence. According to Fey et al. [8] a magnetic field of 10mT does not affect larval growth or body mass; however, they confirmed that this factor influences the rate of yolk sac resorption in rainbow trout (*O. mykiss*) larvae. Studies on the Jaguar cichlid (*P. managuensis*) also showed that exposure to a 5mT magnetic field may negatively affect larval size, as larvae hatched smaller than those in the control group [9]. Conversely, Krylov et al. [6] demonstrated that exposure to magnetic fields affected larval size and body mass in roach (*R. rutilus*). In *P. managuensis*, larvae exposed to magnetic fields of 1mT and 3mT hatched larger than those in the control [9]. Studies by Formicki and Winnicki [5] also showed that generated magnetic fields influence the larvae of rainbow trout (*O. mykiss*) and sea trout (*S. trutta*), with larvae of these species reaching greater size and body mass compared to those not exposed to magnetic fields. Similar results were obtained in the present study on rainbow cichlid larvae exposed to 1mT and 5mT magnetic fields, which hatched larger than those in the control group. In contrast, larvae exposed to a 3mT magnetic field hatched smaller than those incubated without magnetic field exposure.

Interesting findings concerning larval survival and deformities indicate that larvae exposed to magnetic fields in all experimental groups showed higher survival rates than those in the control group not subjected to magnetic exposure. This increase in survival was correlated with a higher incidence of deformities. As survival rates increased, the number of deformed larvae also rose. Similar results were obtained in studies on the Jaguar cichlid (*P. managuensis*), where exposure to magnetic fields likewise increased larval survival. As in the rainbow cichlid, the rise in survival rate in *P. managuensis* was accompanied by a greater number of deformities. It can be assumed that magnetic fields, by enhancing overall survival, may also allow individuals with congenital developmental defects to persist.

## Conclusions

Magnetic fields of selected densities (1mT, 3mT, and 5mT) influenced both the embryonic and post-embryonic development of the rainbow cichlid (*H. multispinosa*). This effect became more pronounced as organogenesis progressed, particularly in the group exposed to the 5mT field. The accelerated developmental rate in this variant resulted in significantly lower egg survival. The magnetic field also affected the larval stage of *H. multispinosa*. Faster embryonic development influenced larval size – embryos exposed to a 5mT magnetic field hatched as the largest individuals with the smallest yolk sacs. However, larvae from this group exhibited the lowest body growth rates in subsequent days. In contrast, larvae in the 3mT group hatched as the smallest and had the second-largest yolk sacs, but showed the fastest growth during the first days of life.

Results concerning larval survival and deformities indicate that larvae exposed to magnetic fields in all experimental variants exhibited higher survival rates than those in the control group. This was correlated with an increased number of deformed larvae – greater survival was accompanied by a higher incidence of body deformities.

The observed effects of magnetic fields on embryonic and larval development may result from a combination of physiological mechanisms. Magnetic exposure could alter cell division rates and metabolic activity, leading to faster yolk utilization and differences in larval size. It may also influence hormonal regulation and growth factors, affecting growth rates and body proportions. Additionally, moderate oxidative stress and changes in cell orientation during organogenesis could explain the increased survival alongside a higher incidence of deformities.

These findings provide valuable insights into the potential use of magnetic fields in ornamental fish aquaculture, where appropriately selected magnetic field densities may positively influence eggs and larvae, reducing losses during rearing. However, to fully elucidate the mechanisms underlying the effects of magnetic fields on the development of *H. multispinosa*, further research is required to examine the impact of this environmental factor on subsequent developmental stages.

- Magnetic fields with intensities of 1mT, 3mT, and 5mT affect the embryonic and post-embryonic development of the honey cichlid (*H. multispinosa*), with the effect increasing during organogenesis and being strongest at 5mT.
- Exposure to the magnetic field alters development rate and larval traits: at 5mT, larvae hatched larger with smaller yolk sacs but exhibited lower body growth in subsequent days, whereas at 3mT, larvae hatched smaller with larger yolk sacs and showed rapid initial growth.
- Magnetic fields increase larval survival compared to the control, although higher survival is associated with a greater number of deformities.
- These results indicate the potential application of magnetic fields in ornamental fish aquaculture to improve larval survival and quality; however, full elucidation of the underlying mechanisms requires further research covering additional stages of fish development.

## Data Availability

The data generated and analysed during the current study are available from the corresponding author upon reasonable request. The data are not publicly available due to the excessive amount of collected data.
